# Global left ventricular function in mice during the development of heart failure

**DOI:** 10.1186/1532-429X-13-S1-P344

**Published:** 2011-02-02

**Authors:** Bastiaan J van Nierop, Elza D van Deel, Dirk J Duncker, Klaas Nicolay, Gustav J Strijkers

**Affiliations:** 1 Eindhoven University of Technology, Eindhoven, Netherlands; 2 Erasmus MC, University Medical Center Rotterdam, Rotterdam, Netherlands

## Introduction

In response to pressure overload the left ventricle (LV) remodels to compensate for the increased workload. Initially this adaptation is beneficial to maintain pump function, but eventually the heart may loose its battle to cope with the increased workload resulting in heart failure (HF). In this study the evolution of global LV function was characterized in a relevant mouse model of LV pressure overload during the development of HF using MRI.

## Methods

**Figure 1:**
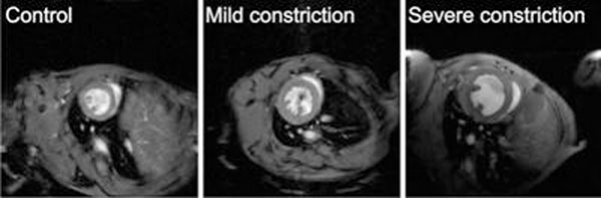
End diastolic images of the LV in the different experimental groups. Note increased wall thickness in mice with a constriction.

C57BL/6 mice (male, age 12 wks) underwent a transverse aortic constriction to induce LV pressure overload. Animals were subjected to a mild (25G, Ø 0.50mm; n=3) or severe constriction (27G, Ø 0.42mm; n=9). Age-matched littermates were used as controls (n=4). MRI measurements acquired at 9.4T were performed starting 1 week post surgery until the age of 22 weeks. Global LV function was characterized from cinematographic MR images (15-18 frames per cardiac cycle) using a respiratory gated and cardiac triggered FLASH sequence (Fig.1). The myocardial wall was segmented semi-automatically using CAAS MRV FARM (Pie Medical Imaging) to obtain cardiac mass, - volume and ejection fraction.

## Results

**Figure 2:**
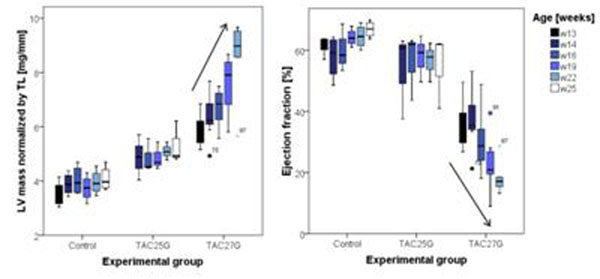
LV mass normalized to tibia length (left) and LV ejection fraction (right) for the different experimental groups as a function of time.

Mice subjected to a mild constriction showed a small increase in LV mass normalized to tibia length as compared to controls (Fig. 2, p<0.01). Ejection fraction was slightly depressed as compared to controls (p<0.05). The severe constriction resulted in a progressive increase in LV mass accompanied by a decline in ejection fraction. In these mice lung edema was observed as indicated by increased lung weight-to-bodyweight ratio 11.9±3.3 mg/g versus 6.3±0.9 mg/g in controls (p<0.05). Moreover, right ventricular EF was depressed, 25.4±20.1% versus 70.8±6.1% in controls (p<0.001).

## Discussion

Mice subjected to a mild constriction of the transverse aorta develop a compensated stage of LV hypertrophy. Mice subjected to a severe constriction show progressive LV hypertrophy accompanied by a decline in LV function. Lung edema and depressed right ventricular EF in this group suggests that these animals develop end stage heart failure. Future work will focus on the imaging of different aspects of adverse remodelling of the LV using MRI.

